# A Study on the Thermal Properties of High-Strength Concrete Containing CBA Fine Aggregates

**DOI:** 10.3390/ma13071493

**Published:** 2020-03-25

**Authors:** In-Hwan Yang, Jihun Park

**Affiliations:** Department of Civil Engineering, Kunsan National University, Jeonbuk 54150, Korea; jhpark3@kunsan.ac.kr

**Keywords:** thermal conductivity, coal bottom ash, unit weight, compressive strength, ultrasonic velocity

## Abstract

The thermal conductivity of concrete is a key factor for efficient energy consumption in concrete buildings because thermal conductivity plays a significant role in heat transfer through concrete walls. This study investigated the effects of replacing fine aggregates with coal bottom ash (CBA) and the influence of curing age on the thermal properties of high-strength concrete with a compressive strength exceeding 60 MPa. The different CBA aggregate contents included 25%, 50%, 75%, and 100%, and different curing ages included 28 and 56 days. For concrete containing CBA fine aggregate, the thermal and mechanical properties, including the unit weight, thermal conductivity, compressive strength, and ultrasonic velocity, were measured. The experimental results reveal that the unit weight and thermal conductivity of the CBA concrete were highly dependent on the CBA content. The unit weight, thermal conductivity, and compressive strength of the concrete decreased as the CBA content increased. Relationships between the thermal conductivity and the unit weight, thermal conductivity and compressive strength of the CBA concrete were proposed in the form of exponential functions. The equations proposed in this study provided predictions that were in good agreement with the test results. In addition, the test results show that there was an approximately linear relationship between the thermal conductivity and ultrasonic velocity of the CBA concrete.

## 1. Introduction

Currently, due to the increasing frequency of extremely hot weather conditions, efficient energy consumption is required in the construction field. In particular, there is an increasing demand for energy-efficient buildings, in which the internal temperature can be optimized [1,2,3]. One of the key factors for optimizing energy efficiency is thermal conductivity. When a building is constructed from materials with high thermal conductivity, a great amount of energy is consumed for cooling and heating [4,5]. To ensure the internal temperature of structures, materials with low thermal conductivity are recommended for constructing concrete structures. Accordingly, concrete with low thermal conductivity is preferable for efficient energy consumption in residential and commercial concrete buildings.

Regarding concrete with low thermal conductivity, some experimental studies have been performed [6,7,8,9,10,11]. Aghdam et al. [6] performed an experimental study to estimate the effects of carbon nanotubes on the thermal conductivity of steel fiber-reinforced concrete. The test results show that the addition and the increasing length of the carbon nanotubes significantly improved the thermal conductivity of steel fiber-reinforced concrete. Wang et al. [7] studied the thermal conductivity of concrete with expanded perlite, which is a porous material, and concluded that the mechanical strength and thermal conductivity of the concrete decreased after the expanded perlite was added to the concrete. Nguyen et al. [8] studied the influence of moisture content and temperature on the thermal properties of lightweight concrete, for which expanded clay, expanded shale, and pumice were used for the fabrication of lightweight aggregates. This study reported that the thermal conductivity of the concrete specimens, including expanded clay, expanded shale, and pumice, showed a great dependence on the moisture content. In addition, Brooks et al. [9] also investigated the effect of different lightweight fillers, including expanded polystyrene beads, dry-expanded thermoplastic microspheres, hollow glass microspheres, and lightweight hollow spheres made of fly ash, on the thermal properties of lightweight cementitious composites. The authors indicated that the thermal properties of lightweight concrete were greatly affected by the type and volume fraction of lightweight filler. Lower-density hollow glass microspheres, expanded polystyrene beads, and dry-expanded thermoplastic microspheres are more suitable for nonstructural thermal insulating components.

The demand for electricity is also an increasingly prevalent issue, and a thermal power plant is one of the methods to supply electricity. However, coal-fired thermal power plants create an enormous amount of bottom ash and fly ash [12,13,14,15,16,17]. Coal bottom ash (CBA) is an industrial waste produced at the bottom of a coal furnace in coal-fired thermal power plants. CBA is a kind of porous material with some advantages, such as low thermal conductivity and low specific density, which can be used in the concrete industry [18,19]. Accordingly, the material properties of CBA concrete have been examined in previous studies [20,21,22]. Mangi et al. [23,24] investigated the effect of CBA on the concrete strength properties under sulfate and chloride environments. Balapour et al. [25] performed an experimental program to investigate the potential use of CBA for the internal curing of concrete systems. They indicated that CBA exhibited a low oven dry-specific gravity, which makes it capable of storing the amount of water needed for concrete internal curing. In addition, Khongpermgoson et al. [26] reported that the compressive strength of concretes mixed with ground CBA and other binders increased with increasing curing age.

However, most previous studies investigated the mechanical properties of CBA concrete with a normal compressive strength of less than 40 MPa, which included CBA as an aggregate replacement. Moreover, few studies have assessed the thermal properties of high-strength CBA concrete. Therefore, to develop concrete with low thermal conductivity for energy efficiency, an experimental study must be performed to investigate the effects of the partial or total replacement of natural aggregates with CBA on the thermal properties of concrete.

In this experimental study, the thermal and mechanical properties of high-strength CBA concrete with a target compressive strength exceeding 60 MPa were investigated. The CBA concrete included 0%, 25%, 50%, 75%, and 100% replacement of natural fine aggregates with CBA aggregates. The unit weight, thermal conductivity, compressive strength, and ultrasonic velocity of the produced concrete were measured. In addition, relationships between the thermal conductivity and the unit weight, compressive strength, and ultrasonic velocity for the CBA concrete were proposed.

## 2. Experimental Program

### 2.1. Materials

**Figure 1:**
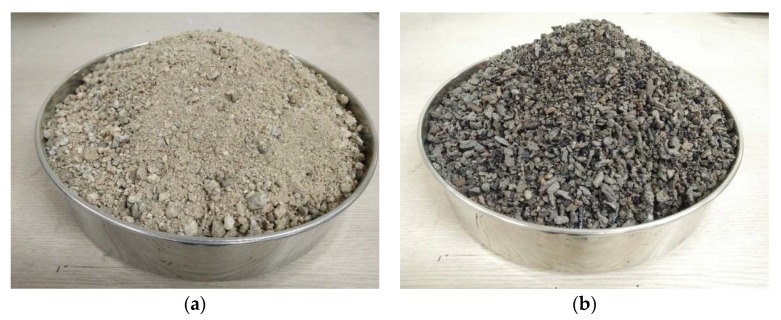
Crushed fine and CBA aggregates. (**a**) Crushed fine aggregate; (**b**) CBA aggregate.

**Figure 2:**
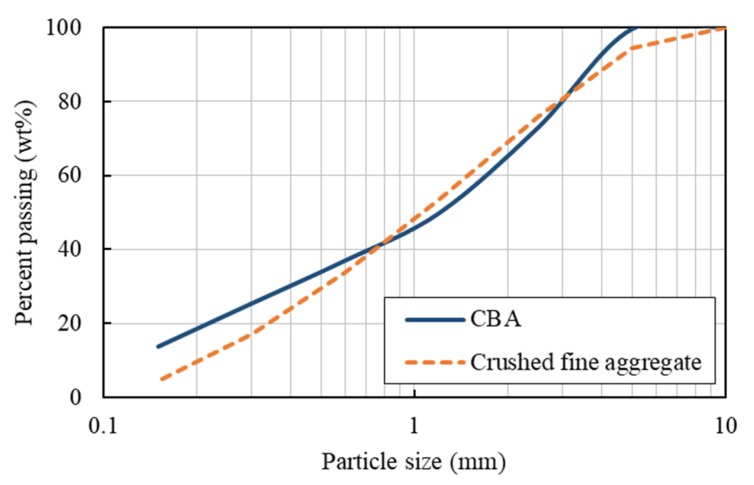
Grading curve of CBA and crushed fine aggregates.

Both the fine and coarse aggregates used in this study were crushed materials. Due to the depletion of natural resources, crushed fine aggregates have been used favorably in Korea. The crushed fine aggregate is shown in Figure 1a. The particle size distribution of the crushed fine aggregate is shown in Figure 2. The minimum and maximum sizes of the coarse aggregates used in this study were 5 and 20 mm, respectively. The density, water absorption, and fineness modulus of the crushed fine and coarse aggregates used are shown in Table 1. The densities of the fine and coarse aggregates were 2.60 and 2.61 g/cm^3^, respectively, and the water absorption of the fine and coarse aggregates were 0.69 and 1.44, respectively.

The CBA was collected from a thermal power plant company (Korea South-East Power Co., Ltd, Yeongheung Power Division, Yeongheung, Korea). The chemical components of the CBA used were determined through energy-dispersive spectroscopy (EDS, Hitachi High-Technologies Corporation, Tokyo, Japan), and the analysis results are shown in Table 2. The three major components in the CBA were SiO_2_, Al_2_O_3_, and Fe_2_O_3_, which had contents of 60.03%, 20.25%, and 9.80%, respectively, thereby comprising greater than 90% of the CBA.

**Figure 3:**
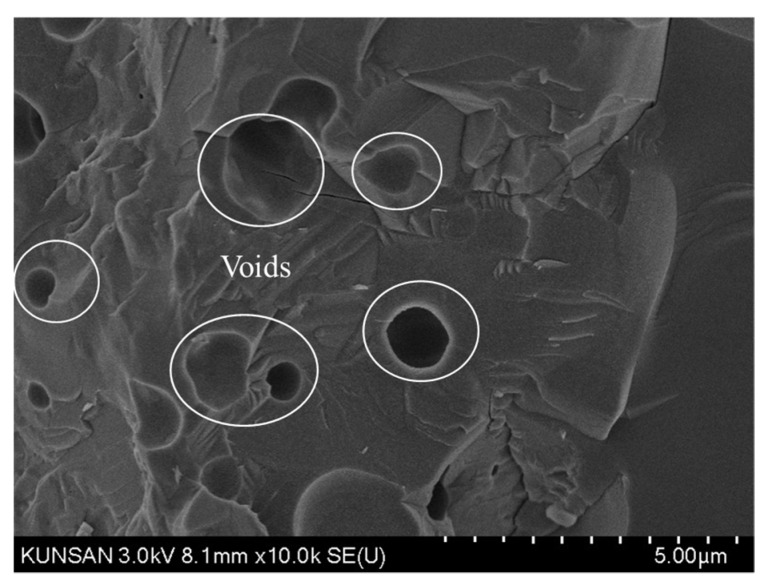
SEM image of CBA aggregate.

The CBA aggregate was screened to remove particles greater than 5.0 mm and to retain particles greater than 0.15 mm. The CBA used in this study is shown in Figure 1b, and the particle size distribution of the CBA is also presented in Figure 2. A scanning electron microscopy (SEM) image of the CBA is given in Figure 3, and the image shows the presence of voids in the CBA particles. As shown in Table 1, the density of the CBA was smaller than that of the crushed fine aggregate, which were 1.84 and 2.60 g/cm^3^, respectively. On the other hand, the CBA water absorption was much higher than that of the crushed fine aggregate, which were 6.87% and 0.69%, respectively.

Ordinary Portland cement (OPC) used in this study was type I in accordance with KS L 5201 [27]. The specific gravity of the OPC used was 3.15, and the chemical components of the OPC are shown in Table 2. To enhance the workability and reduce the water-cement ratio of the CBA concrete, a superplasticizer with a dosage of 3.6 kg/m^3^, which corresponded to 0.6% of the weight of the OPC, was used.

### 2.2. Mixing Proportions

The mixing proportions of the control concrete and CBA concrete are provided in Table 3. A concrete mix was designed with a target compressive strength of 60 MPa at a curing age of 28 days. In the experimental study, five different series of concrete mixtures were prepared with various percentages of CBA as crushed fine aggregate replacement. The crushed fine aggregate in the concrete was replaced with CBA at five different volume fractions of 0%, 25%, 50%, 75%, and 100%, and the corresponding CBA concrete mixtures were named CBA00, CBA25, CBA50, CBA75, and CBA100, respectively. The amounts of cement and coarse aggregate were constant for each concrete mixture at 595.0 and 878.5 kg/m^3^, respectively. A water-cement ratio of 0.3 was applied in all of the concrete mixtures.

### 2.3. Specimen Preparation and Test Procedures

All of the concrete specimens were fabricated in a laboratory mixer. Cylindrical specimens with dimensions of 100 mm × 200 mm were fabricated to determine the unit weight, thermal conductivity, and compressive strength of the different mixtures. After casting the concrete, the concrete specimens were covered with plastic wrap and moist-cured for one day. Thereafter, the specimens were demolded at an age of 24 ± 1 h and then cured under submersed conditions at 23 ± 2 °C in a water tank until the ages of 28 and 56 days after the casting of concrete.

The unit weight, compressive strength, thermal conductivity, and ultrasonic velocity of the CBA concrete were measured at curing ages of 28 and 56 days. Both end surfaces of the cylinders for compressive strength tests were ground before implementing each experiment.

The unit weight (bulk density) of hardened CBA concrete was measured by using the cylindrical specimens after curing for 28 and 56 days, respectively. The unit weight was determined by dividing the mass of the cylindrical specimen by the volume of the specimen.

The compressive strength of the cylindrical specimens was tested with a universal testing machine (UTM). Loading was applied under displacement control using a UTM with a capacity of 2000 kN. The mean values of three specimens were recorded to obtain the material properties of the concrete.

There are several testing methods and their related devices for the measurement of the thermal conductivity of concrete. First, the thermal conductivity test can be carried out in accordance with ASTM D 5334-05 [28]. The ASTM method is based on the concept that the temperature rise in the heat source depends on the thermal conductivity of the medium into which it is inserted. The probe consists of a heating wire and a temperature measuring unit, and it should be inserted into a hole drilled in the concrete specimen. Similarly, Kim et al. [29] also used the two linear parallel probe (TLPP) method to determine the thermal conductivity of concrete. For the TLPP method, two probes are inserted into two parallel holes drilled in the specimen, where one probe is used as a heating source and the other is used as a temperature sensor.

The transient plane source (TPS) method has been explained in detail by Gustafsson [30] and Log and Gustafsson [31], and its consideration was summarized by He [32]. For the TPS method, the probe is sandwiched between the cast sides of two specimens or the cut faces of two elements of a concrete specimen, whereas probe rods are inserted into holes in the concrete specimen when using the ASTM method and TLPP method. The TPS method has been widely used to measure the thermal conductivity of solid materials such as concrete [33,34].

**Figure 4:**
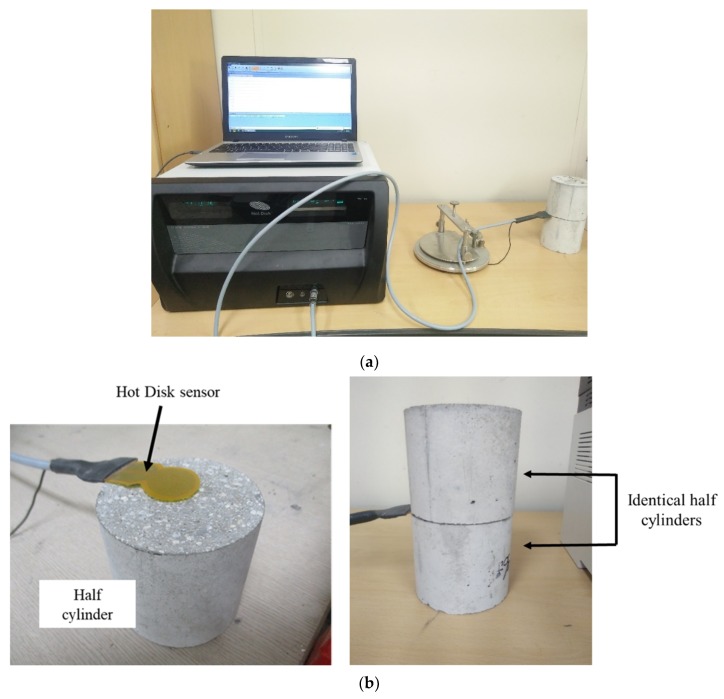
Test setup for the thermal conductivity measurements. (**a**) Transient plane source (TPS) measurement system; (**b**) concrete specimens used for the thermal conductivity experiments.

In this study, the measurement of the thermal conductivity was based on the TPS method. The thermal conductivity of the CBA specimens was measured using a TPS1500 testing device supplied from Hot Disk Ltd. (Gothenburg, Sweden) as shown in Figure 4a. In the TPS method, to ensure that the sensor was exposed to fine and coarse aggregates and cement paste, the cylindrical specimens were cut into two halves at the middle section of the cylinder, as shown in Figure 4a, and then the sensor was sandwiched between the two half cylinders, as shown in Figure 4b. The cut surfaces had planeness to ensure contact between the concrete specimen and the sensor.

The sensor contained a nickel double spiral that applied a heating pulse to the specimen. The concrete specimen was controlled to satisfy thermal equilibrium before the measurements. After a thermal equilibrium time of at least 90 min under laboratory temperature conditions, measurements were made with an applied heating power of 0.3 W. Three measurements were taken for each specimen to ensure accurate test results.

## 3. Test Results and Discussion

### 3.1. Properties of Fresh Concrete

**Figure 5:**
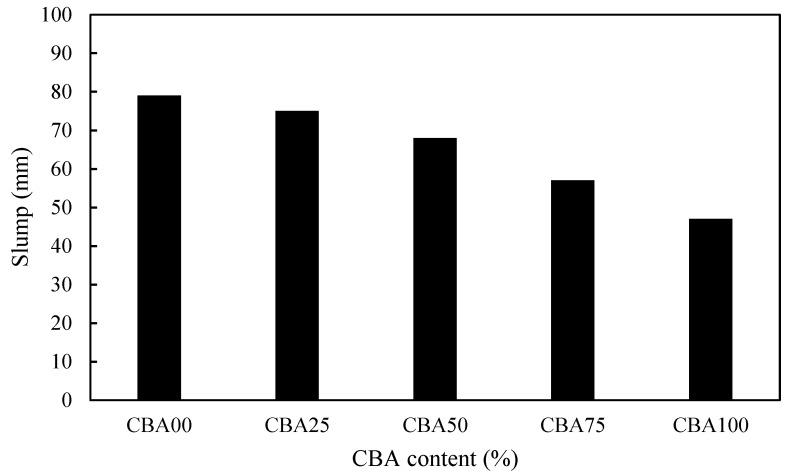
Slump test results.

To investigate the workability of the fresh concrete, a slump test was performed. The slump test results are shown in Figure 5. The slump of the CBA concrete mixtures decreased as the CBA fine aggregate content increased. The slumps of CBA concrete mixtures CBA25, CBA50, CBA75, and CBA100 were 75, 68, 57, and 47 mm, respectively, whereas that of the control concrete mixture was 79 mm. The decrease in the workability of concrete is mainly due to the irregular shapes and the increase in the surface area of the aggregates used in concrete. The use of CBA as fine aggregates affected the concrete texture, which had more irregular and porous particles than the control concrete. Therefore, the friction between particles in CBA concrete increased the obstruction of the workability of the fresh concrete and then led to a decrease in the slump of the CBA concrete.

### 3.2. Unit Weight

**Figure 6:**
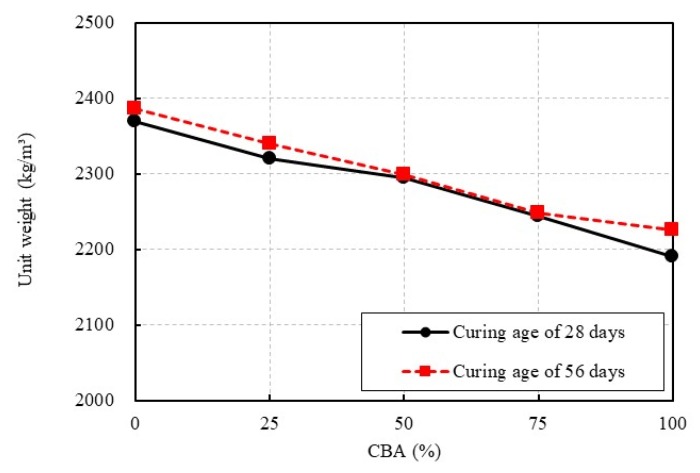
Unit weight test results.

The unit weights of the concrete specimens with different CBA replacement ratios are shown in Figure 6. The figure indicates that the unit weight of the CBA concrete decreased as the CBA replacement ratio increased in the concrete, which was the expected response. At a curing age of 28 days, the unit weight of the control mixture CBA00 was 2370.2 kg/m^3^, whereas the unit weight of the mixture containing 100% CBA fine aggregate decreased to as low as 2190.2 kg/m^3^. Specifically, the unit weights of the CBA concrete mixtures CBA25, CBA50, CBA75, and CBA100 were 2.1%, 3.2%, 5.3%, and 7.6% less than that of the control mixture, respectively. This decrease in the unit weight of the CBA concrete mixtures occurred because the unit weight of CBA fine aggregate was lower than that of crushed fine aggregate, as shown in Table 1. The porosity of each CBA concrete specimen was estimated by using the mercury intrusion porosity (MIP) method. After performing the compressive strength test, the crushed concrete specimen was broken into small samples to be placed in the MIP dilatometer. For the small samples, coarse aggregates were eliminated from the samples. Then, the MIP test was carried out by using the concrete piece samples. The porosity in the sample consisted of the contribution from cement paste and that from CBA fine aggregates. Accordingly, it could be considered that the porosity from the MIP test was affected by the CBA fine aggregate contents under the conditions of a constant water-cement ratio and the use of the same type of cement. The MIP test results in the present study show that the porosity of mixture CBA100 was higher than that of the reference mixture (CBA00). Specifically, the porosities for mixtures CBA00, CBA25, CBA50, CBA75, and CBA100 at a curing age of 28 days were 8.5%, 9.6%, 10.4%, 12.3%, and 15.6%, respectively. Thus, the porosity increased as the CBA aggregate content increased.

At a curing age of 56 days, the unit weight of the control mixture was 2386.5 kg/m^3^, whereas the unit weight of mixture CBA100 decreased to as low as 2225.7 kg/m^3^. Specifically, the unit weights of the CBA concrete mixtures CBA25, CBA50, CBA75, and CBA100 were 2.0%, 3.7%, 5.8%, and 6.7% less than that of the control mixture, respectively.

Figure 6 also compares the measured unit weights of the concrete specimens at different curing ages. At a curing age of 56 days, the unit weights of the concrete with 0%, 25%, 50%, 75%, and 100% CBA replacement of crushed fine aggregate were 0.7%, 0.9%, 0.2%, 0.1%, and 1.6% higher than the corresponding values at a curing age of 28 days, respectively; hence, these increases were insubstantial.

### 3.3. Thermal Conductivity

**Figure 7:**
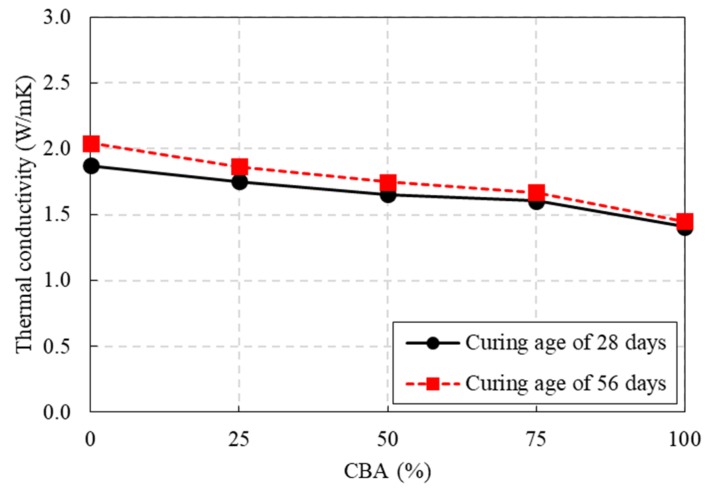
Thermal conductivity test results.

The thermal conductivities of the concrete specimens with different CBA contents are presented in Figure 7. The thermal conductivities of CBA concrete decreased as the CBA content increased at a curing age of 28 days. The thermal conductivities of the CBA concrete mixtures CBA25, CBA50, CBA75, and CBA100 were 6.4%, 11.7%, 14.2%, and 22.5% less than that of the control concrete mixture CBA00 (1.87 W/mK), respectively. At a curing age of 56 days, the thermal conductivity of 1.45 W/mK in the CBA concrete mixture CBA100 was 31.2% less than the value of 2.04 W/mK in the control concrete mixture. The thermal conductivity of the CBA concrete with a 100% CBA content was significantly less than that of the control concrete mixture. It is known that thermal conductivity highly depends on the pore structure of the concrete, and subsequently the density of the concrete. Hence, the pore structure was one of the key elements affecting thermal conductivity [10,35]. As already discussed in the previous section, the porosity of the CBA concrete specimen for each mixture increased as the CBA aggregate content increased. As the CBA content increased, the total porosity increased, so the thermal conductivities of the concrete decreased. For this reason, the observed decline in thermal conductivity could be explained by the increase in the CBA aggregate content in the concrete.

A decrease in the thermal conductivity of the CBA concrete would increase the thermal insulation provided by the concrete and reduce the heating and cooling costs for buildings constructed from these materials. Therefore, the test result of the thermal conductivities for CBA concrete in this study implies that CBA could be utilized to fabricate high-strength concrete with low thermal conductivity for efficient energy consumption.

The effect of curing age on the thermal conductivity of the CBA concrete specimens is also shown in Figure 7. When the concrete curing age increased from 28 to 56 days, the thermal conductivities of the concrete with CBA contents of 0%, 25%, 50%, 75% and 100% CBA increased by 9.2%, 6.5%, 5.7%, 4.0%, and 3.1%, respectively. With the increase in curing age, the pores in the concrete matrix were filled by hydration products and calcium silicate hydrate (CSH) gel [36]. Heat is transferred faster in solid materials than in porous materials. Therefore, the thermal conductivity for the well packed concrete specimens at a curing age of 56 days will be higher than that at a curing age of 28 days.

**Figure 8:**
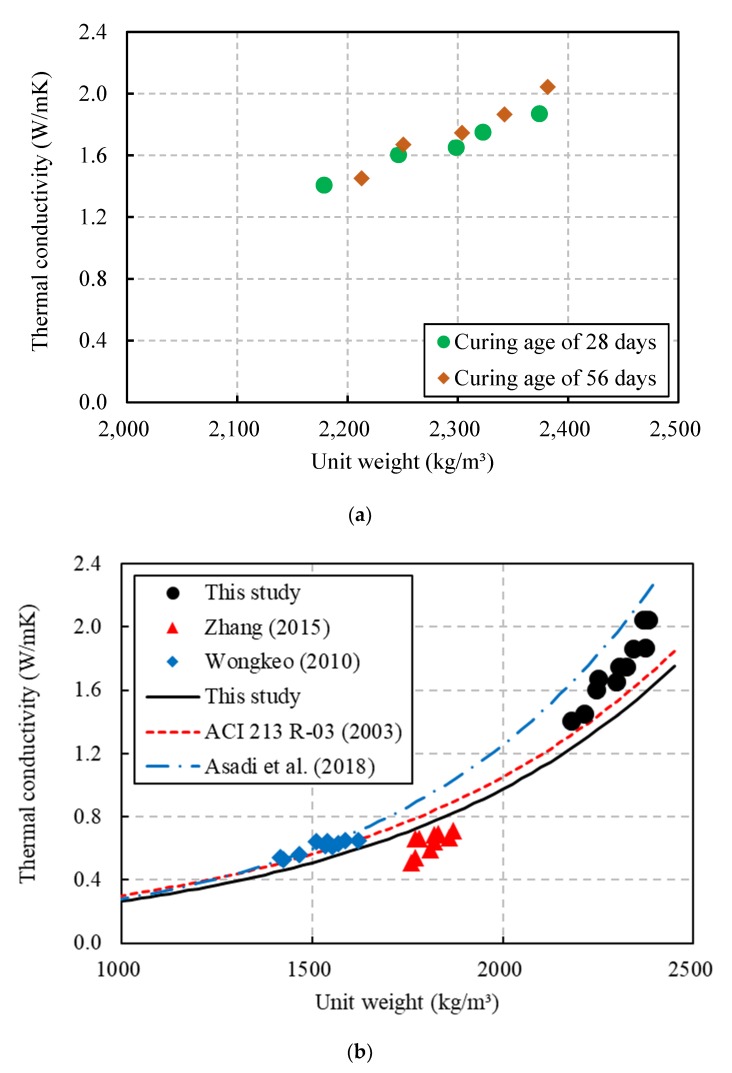
Relationship between the thermal conductivity and the unit weight. (**a**) Test results in this study; (**b**) comparison of the predictions in this study and previous studies

The relationship between the thermal conductivity and the unit weight of the CBA concrete with two different curing ages is shown in Figure 8a. As the unit weights of the CBA concrete increased, the thermal conductivity of the CBA concrete increased. Moreover, the thermal conductivity of the CBA concrete is nearly linearly proportional to the unit weight. This phenomenon occurred because the substitution of CBA as fine aggregate increased the porosity in the concrete, thereby reducing the thermal conductivity and unit weight of the CBA concrete. The smallest unit weight was nearly consistent with the smallest thermal conductivity of CBA concrete.

Asadi et al. [11] proposed Equation (1) to predict the thermal conductivity of CBA concrete by using the unit weight. Their proposed equation was an exponential function that was derived based on the test data of thermal conductivities of lightweight concrete available in the literature. The test data used in their equation did not contain only CBA but also pumice, expanded polystyrene, and expanded perlite.

(1) $$k=0.0625e^{0.0015\rho }$$

where k is the thermal conductivity (W/mK) and *ρ* is the unit weight of the CBA concrete (kg/m^3^).

ACI committee 213 R-03 [37] proposed Equation (2) to estimate the thermal conductivity of lightweight concrete.

(2) $$k=0.0864e^{0.00125\rho }$$

Wongkeo [35] and Zhang et al. [38] carried out an experimental program on CBA concrete, and their studies indicated that the thermal conductivities had a close relation with the unit weight. This study proposes Equation (3), which is based on the test results from the present study and the results from Wongkeo [35] and Zhang et al. [38]. A comparison of the three equations is also shown in Figure 8b.

(3) $$k=0.0725e^{0.0013\rho }$$

The equation from Asadi et al. [11] overestimated the thermal conductivities because it was derived based on concrete including different kinds of lightweight aggregates. In contrast, the equation proposed in this study provides predictions that are in close agreement with the test results of the CBA concrete.

### 3.4. Compressive Strength

**Figure 9:**
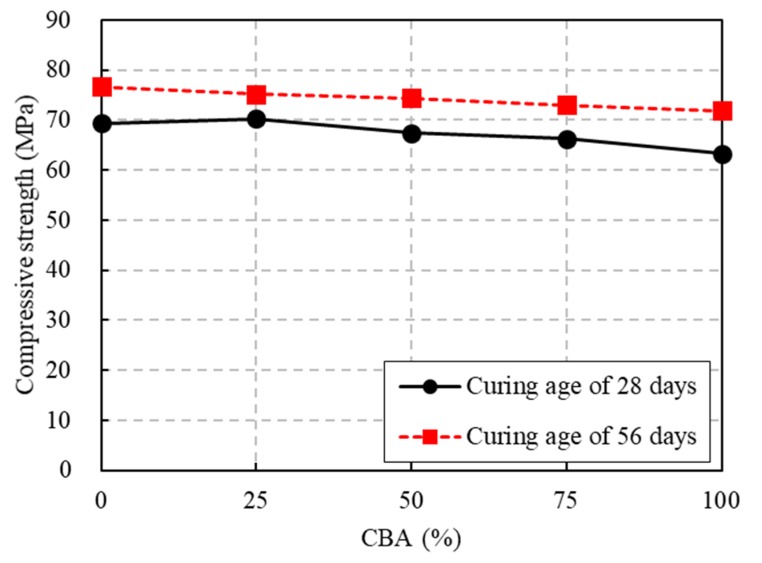
Compressive strength test results.

The test results of the compressive strength of the concrete specimens with different CBA contents are shown in Figure 9. At a curing age of 28 days, the compressive strength of the CBA mixtures decreased as the CBA replacement increased. The compressive strength values of CBA concrete mixtures CBA50, CBA75, and CBA100 were 3.0%, 4.6%, and 8.8% less than that of the control concrete mixture (CBA00), respectively. However, the compressive strength of the CBA concrete mixture CBA25 was only 1.2% higher than that of the control concrete mixture. At a curing age of 56 days, the compressive strength values of the CBA concrete mixtures CBA25, CBA50, CBA75 and CBA100 were 2.0%, 3.0%, 4.8%, and 6.2% less than that of the control concrete mixture (CBA00), respectively. This compressive strength loss could be explained by the increase in the porosity of the concrete. These pores might have an adverse influence on the compressive strength of the CBA concrete specimens [39].

Figure 9 also illustrates the effects of curing ages of 28 and 56 days on compressive strength. At a curing age of 56 days, the concrete compressive strength values with CBA contents of 0%, 25%, 50%, 75%, and 100% were 110.4%, 106.9%, 110.4%, 110.2%, and 113.6% of the corresponding values at a curing age of 28 days, respectively. The substantial increase in the compressive strength of the CBA concrete mixtures after curing for 56 days might result from the pozzolanic reaction of the CBA. According to the study of Abdulmatin et al. [36], due to the pozzolanic activity, secondary CSH and calcium aluminate hydrate (CAH) form; therefore, the porosity of the concrete matrix is filled with these materials. In addition, Ca(OH)_2_ is transformed into CSH. These phenomena are why the concrete compressive strength increased with the increase in curing age.

**Figure 10:**
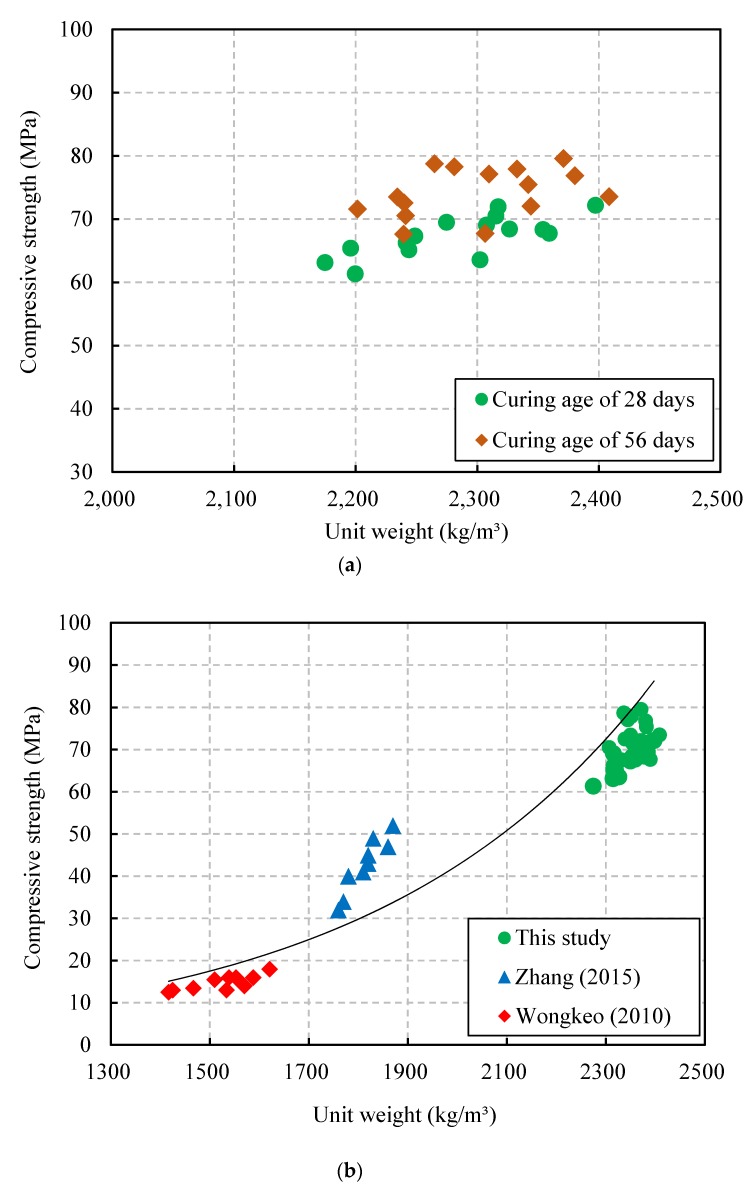
Relationship between the compressive strength and the unit weight. (**a**) Test results in this study; (**b**) relationship based on the results in this study and previous studies.

The relationship between the compressive strength and the unit weight of the CBA concrete under two different curing ages is presented in Figure 10a. The compressive strength of the CBA concrete has a nearly direct relationship with the unit weight of the CBA concrete. The compressive strength of the CBA concrete increased as the unit weight of the CBA concrete increased. This phenomenon occurred because both the unit weight and the compressive strength of the CBA concrete were affected by the replacement of a stronger material (crushed sand) with a weaker material (CBA) and the increase in pore volume in the CBA concrete, as described in the previous section.

The relationship between the compressive strength and the unit weight of the CBA concrete, based on the test results in this study and in previous studies [35,38], is shown in Figure 10b. The proposed exponential equation for predicting the relationship between the compressive strength and the unit weight of the CBA concrete is expressed as follows:

(4) $$f_{c}=1.217e^{0.0018\rho }$$

where $f_{c}$ is the compressive strength (MPa) and ρ is the unit weight (kg/m^3^).

This equation underestimated the compressive strength values when the unit weight ranged from approximately 1700 to 1900 kg/m^3^, whereas it overestimated the compressive strength values when the unit weight ranged from approximately 2300 to 2400 kg/m^3^.

**Figure 11:**
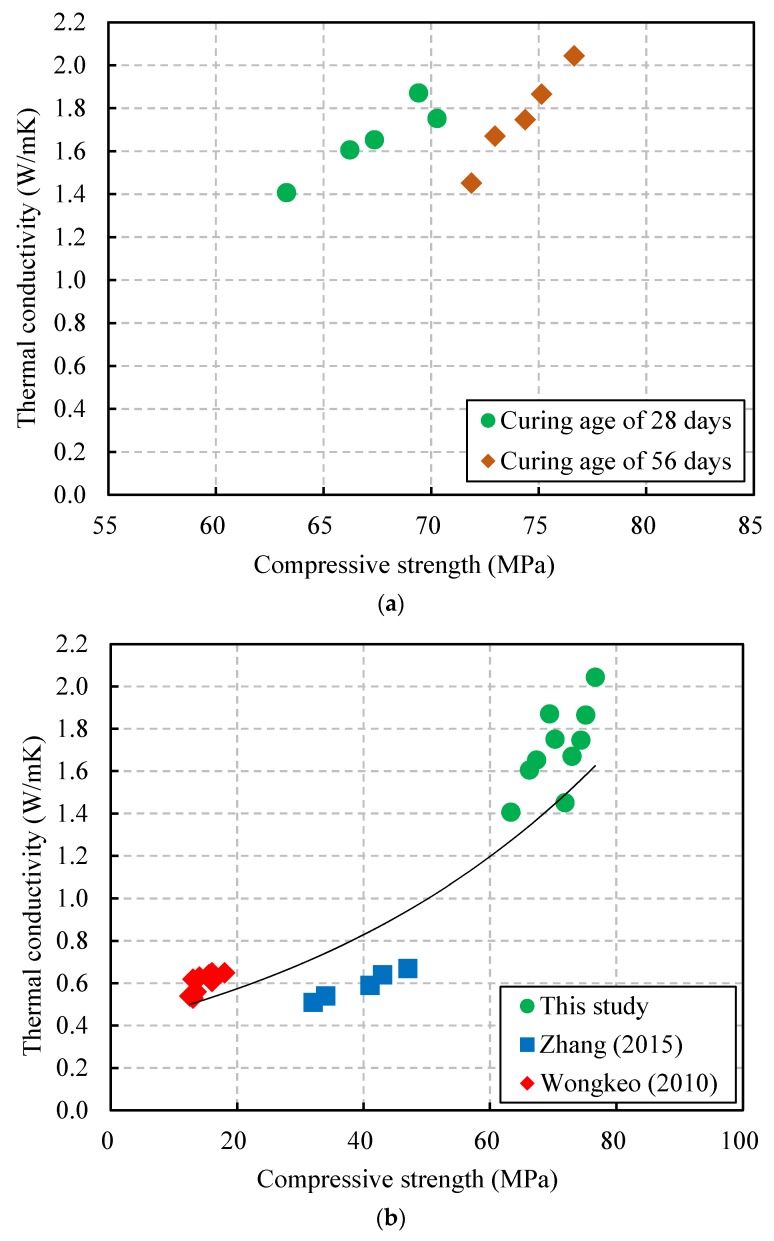
Relationship between the thermal conductivity and the compressive strength. (**a**) Test results in this study; (**b**) relationship based on the results in this study and previous studies.

In addition, the relationship between the thermal conductivity and the compressive strength is shown in Figure 11a. The figure shows that this relationship tendency was similar to that between the thermal conductivity and the unit weight of the CBA concrete. The compressive strength of the CBA concrete was affected by the unit weight. Therefore, the thermal conductivity had a close relationship with the compressive strength of the CBA concrete. In this study, the thermal conductivity of the CBA concrete varied from 1.41 to 2.04 W/mK when the compressive strength ranged from 63.3 to 76.7 MPa. Albayrak et al. [40] also reported that the compressive strength values and thermal conductivities of lightweight concrete decreased with decreasing density.

Moreover, the relationship between the thermal conductivity and the compressive strength, which is based on the test results in this study and in previous studies [35,38], is shown in Figure 11b. The exponential equation form used to predict the relationship between the thermal conductivity and the unit weight of CBA concrete was also applied to predict the relationship between the thermal conductivity and compressive strength as follows:

(5) $$k=0.3976e^{0.0184f_{c}}$$

where k is the thermal conductivity (W/mK) and $f_{c}$ is the compressive strength (MPa).

Overall, the thermal conductivity test results in the figure had some deviations with various CBA concrete compressive strength values. Therefore, the equation overestimated the thermal conductivities when the measurements of compressive strength ranged from approximately 30 to 50 MPa, whereas it underestimated the thermal conductivities when the measurements of compressive strength ranged from approximately 60 to 80 MPa.

### 3.5. Ultrasonic Velocity

**Figure 12:**
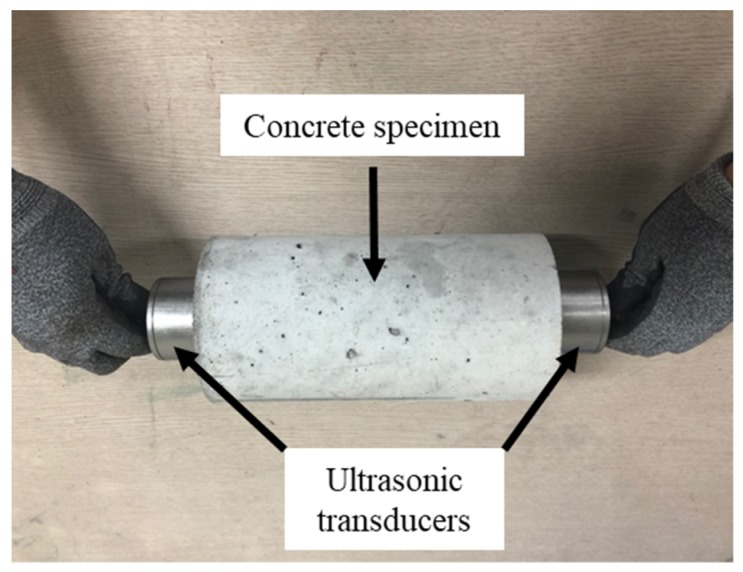
Measurement of ultrasonic velocity.

An ultrasonic pulse velocity test was carried out to assess the material characterization of the CBA concrete. The measured quantity of this experiment was the travel time of the ultrasonic pulse between the transducers that were held on each surface of a concrete specimen, as shown in Figure 12; the pulse velocity was calculated by dividing the distance between the transducers by the travel time.

According to the ASTM C597-09 [41], the frequency of the ultrasonic pulse test procedure should be greater than 50 kHz to achieve accurate transit-time measurements and greater sensitivity for the short measured path. In the previous studies by Ashrafian et al. [42] and Nik et al. [43], the pulse frequency of 54 kHz was applied for measuring the ultrasonic velocity of the concrete cubes of 100 mm. In this study, therefore, an ultrasonic instrument with a frequency of the transducers of 54 kHz was used to measure the ultrasonic velocity.

**Figure 13:**
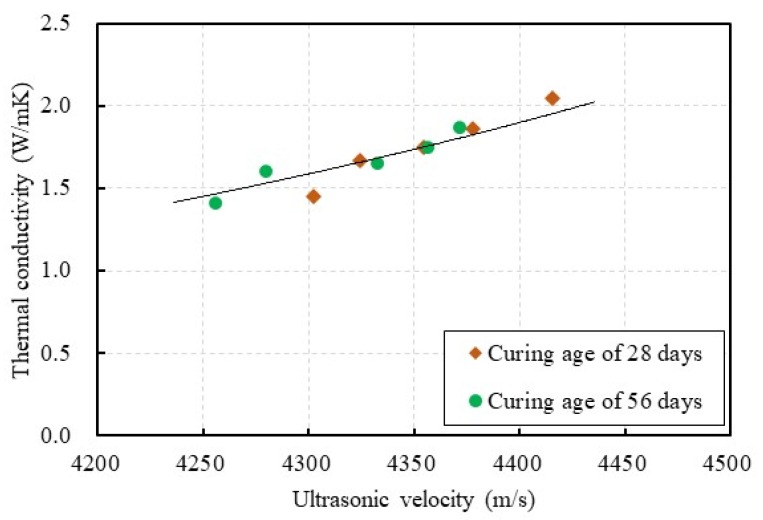
Relationship between the thermal conductivity and the ultrasonic velocity.

The relationship between the thermal conductivity and the ultrasonic velocity is shown in Figure 13. In this study, the thermal conductivity of the concrete specimen ranged from 1.41 to 2.04 W/mK when the ultrasonic velocity ranged from 4256 to 4415 m/s. The test results in this study show that there was an approximately linear relationship between the thermal conductivity and the ultrasonic velocity of CBA concrete. Solid materials transfer sound faster than porous materials. Higher ultrasonic velocity indicates that the concrete has greater continuity, whereas lower ultrasonic velocity indicates that the concrete contains more voids and defects (e.g., cracks). The thermal conductivity and ultrasonic velocity are mainly dependent on the density of the concrete [44]. The addition of the CBA aggregate reduced the density of the CBA concrete, which resulted in a reduction in both the thermal conductivity and ultrasonic velocity.

## 4. Conclusions

In this research, an experimental study was performed to investigate the thermal conductivity of CBA concrete with a compressive strength exceeding 60 MPa. The research findings are summarized as follows:

- The unit weight of CBA concrete decreased as the replacement of CBA as fine aggregate increased. This decrease in the unit weight of the CBA concrete mixtures occurred because the CBA had a lower unit weight and a higher porosity than the crushed fine aggregate.
- The thermal conductivity of the CBA concrete was highly dependent on the CBA content. In addition, overall, the thermal conductivity of the CBA concrete increased as the curing age increased. When the curing age increased from 28 to 56 days, the thermal conductivity of the concrete increased by 3.1~6.5%.
- The relationship between the thermal conductivity and the unit weight of the CBA concrete was modeled with an exponential function. The results indicate that the equation proposed in this study provides predictions that are in good agreement with the test results.
- The compressive strength of the CBA concrete decreased as the CBA content in the concrete increased. In addition, an equation relating the thermal conductivity of the CBA concrete to the compressive strength was proposed. The equation overestimates the thermal conductivity of moderate-strength concrete (30~50 MPa), whereas it underestimates the thermal conductivity of high-strength concrete (60~80 MPa) because the test results used as a basis for the equation have some deviations.
- The ultrasonic velocity of the CBA concrete decreased as the amount of CBA fine aggregate in the concrete increased. Moreover, the test results show that there was an approximately linear relationship between the thermal conductivity and ultrasonic velocity of the CBA concrete.

## Figures and Tables

**Table 1:** Physical properties of fine, coarse and coal bottom ash (CBA) aggregates.

	Property	Fineness Modulus	Water Absorption (%)	Density (g/cm^3^)
Material				
Crushed fine aggregate		3.17	0.69	2.60
Coarse aggregate		6.77	1.44	2.61
CBA		3.83	6.87	1.84

**Table 2:** Chemical components of CBA and OPC.

Component	CBA (%)	OPC (%)
SiO_2_	60.03	31.90
Al_2_O_3_	20.25	8.97
Fe_2_O_3_	9.80	0.87
CaO	5.58	46.95
Na_2_O	1.95	0.38
MgO	1.44	3.25
K_2_O	0.95	0.96
SO_3_	-	5.25

**Table 3:** Mixing proportions of the CBA concrete.

Mixtures	CBA Content (%)	W/C	Water	Unit Content (kg/m^3^)				
				Cement (OPC) ^a^	Coarse Aggregate	Crushed Fine Aggregate	CBA	Superplasticizer (0.6% × Cement)
CBA00	0	0.3	178.5	595.0	878.5	663.0	0.0	3.6
CBA25	25	0.3	178.5	595.0	878.5	497.2	117.7	3.6
CBA50	50	0.3	178.5	595.0	878.5	331.5	235.3	3.6
CBA75	75	0.3	178.5	595.0	878.5	165.7	353.0	3.6
CBA100	100	0.3	178.5	595.0	878.5	0.0	470.7	3.6

^a^ OPC: ordinary Portland cement.

## Data Availability

The data used to support the findings in this study are available from the corresponding author upon request.
